# The Role of Cue Utilization and Cognitive Load in the Recognition of Phishing Emails

**DOI:** 10.3389/fdata.2020.546860

**Published:** 2020-09-24

**Authors:** George Nasser, Ben W. Morrison, Piers Bayl-Smith, Ronnie Taib, Michael Gayed, Mark W. Wiggins

**Affiliations:** ^1^School of Psychology, Charles Sturt University, Bathurst, NSW, Australia; ^2^Department of Psychology, Macquarie University, Sydney, NSW, Australia; ^3^Data 61, Commonwealth Scientific and Industrial Research Organisation (CSIRO), Canberra, ACT, Australia

**Keywords:** phishing, decision-making, cue utilization, security, expertise, cognitive load

## Abstract

Phishing emails represent a major threat to online information security. While the prevailing research is focused on users' susceptibility, few studies have considered the decision-making strategies that account for skilled detection. One relevant facet of decision-making is cue utilization, where users retrieve feature-event associations stored in long-term memory. High degrees of cue utilization help reduce the demands placed on working memory (i.e., cognitive load), and invariably improve decision performance (i.e., the information-reduction hypothesis in expert performance). The current study explored the effect of cue utilization and cognitive load when detecting phishing emails. A total of 50 undergraduate students completed: (1) a rail control task; (2) a phishing detection task; and (3) a survey of the cues used in detection. A cue utilization assessment battery (EXPERTise 2.0) then classified participants with either higher or lower cue utilization. As expected, higher cue utilization was associated with a greater likelihood of detecting phishing emails. However, variation in cognitive load had no effect on phishing detection, nor was there an interaction between cue utilization and cognitive load. Further, the findings revealed no significant difference in the types of cues used across cue utilization groups or performance levels. These findings have implications for our understanding of cognitive mechanisms that underpin the detection of phishing emails and the role of factors beyond the information-reduction hypothesis.

## Introduction

### The Phishing Problem

The accessing of sensitive and personal information by cybercriminals is one of the five most serious risks facing the world today (World Economic Forum, 2019). The most common way that criminals access such information is through phishing attacks. Phishing attacks involve the use of technical mediums, such as emails whereby the sender attempts to engineer a seemingly authentic communication that induces the recipient to open a malicious link or download a malicious attachment (Workman, 2008).

Cybercriminals prefer to target the email user directly because they are seen as the weakest link in the information security chain (Herzberg, 2009). Indeed, when in the workplace email users are often under time pressure, working to deadlines, and completing multiple tasks at any given time. The additional strain placed on information processing is seen as a contributing factor negatively impacting their judgement and decision-making capacity (Wang et al., 2012).

The largest phishing-based data breach occurred at Yahoo in 2013. The attack resulted in the loss of the names, birthdates, phone numbers, passwords, security questions, and backup email address of all three billion customers and wiped 350 million US dollars off their sale price to Verizon Commination (Perlroth, 2017). In response to the rising threat of cyberattacks, organizations around the world spend 114 billion US dollars each year on cybersecurity products and services (Moore and Keen, 2018). However, despite such efforts, between 10 and 20% of phishing emails will still reach a user's inbox (Wombat Security Technology, 2019). In large organizations, this can amount to thousands of such emails arriving in employees' inboxes each year, each with the potential to seriously disrupt productivity and damage reputation (Vergelis et al., 2019).

Over the last decade, a broad range of approaches have explored the reasons why certain users are more susceptible than others to cyberattacks (Vishwanath et al., 2011; Yan and Gozu, 2012; Jones et al., 2015, 2019; Butavicius et al., 2016; Williams et al., 2018; Ayaburi and Andoh-Baidoo, 2019). However, little research has explored the cognitive-perceptual strategies that users employ when making *successful* decisions about an email's legitimacy, such as the skilled use of cue-based associations (Wiggins and O'Hare, 2003; Morrison et al., 2013a,b; Morrison and Morrison, 2015; Wiggins, 2015; Johnston and Morrison, 2016). In the context of phishing detection, cue utilization is presumed to involve an individual's capacity to recognize features within an email that signal (often rapidly and unconsciously) an attempt to deceive. For instance, when tracking emails users' eye-movements during an experimental phishing detection task, Neupane et al. (2015) found that those users who performed worst on the task spent significantly less time attending to highly diagnostic cues (e.g., the URL), and more time looking at irrelevant ones (e.g., the login field).

It is believed that those proficient in the diagnosis of phishing emails will automatically recognize features that cue useful patterns from memory, and which “trigger” the rapid retrieval of a plausible response (i.e., a process of recognition-primed decision-making; Klein, 1993). The timely recognition of these patterns will invariably reduce the demands placed on working memory, with attentional resources being deployed selectively to task-relevant features in the environment (Haider and Frensch, 1999). Thus, when decision-makers possess a greater capacity for cue utilization, they have additional cognitive resources to respond to incoming demands (Ericsson and Lehmann, 1996; Brouwers et al., 2017). This implies that greater levels of cue utilization may “buffer” against the usually deleterious impacts of increased cognitive load by reducing the amount of information in the environment that needs to be processed. Such a strategy may be particularly useful in the context of phishing detection, since it is a process often engaged in tandem with other complex, resource-demanding tasks. Consistent with an information-reduction hypothesis (Haider and Frensch, 1999), behavior associated with relatively higher cue utilization is likely to be associated with higher levels of task performance under increasing cognitive load (e.g., that arising from an increase in task complexity).

### Study Aims

The current study was designed to test the impact of cue utilization and cognitive load on email users' ability to detect phishing emails under conditions of low, moderate, and high cognitive load. In this article we extend on the findings summarized in Nasser et al. (2020) incorporating a more detailed description of our methodology, as well as additional analyses exploring the potential relationships between cue utilization and cue typology.

Conducted in a laboratory setting, participants were asked to manage their attention between a rail control task on one computer screen (Brouwers et al., 2017), and a phishing detection task on another computer screen. Upon completion, participants also completed task response feedback items to understand what cues they relied on when making their decisions during the phishing detection task.

Finally, behavior associated with the utilization of cues was assessed using the Expert Intensive Skills Evaluation (EXPERTise 2.0) assessment tool (Loveday et al., 2014). EXPERTise 2.0 comprises five tasks, each of which is designed to evaluate behavior associated with the application of cue-based associations in memory. Since cues are task-specific, an edition of the tool was developed through the consultation with cybersecurity experts and incorporated features associated with phishing emails. EXPERTise 2.0 has been used previously to delineate behavior associated with higher and lower cue utilization in fields as diverse a pediatric intensive care (Loveday et al., 2013b), software engineering (Loveday et al., 2014), and football coaching (Yee et al., 2020).

### Hypotheses and Research Questions

**Hypothesis one**. Email users' performance on the phishing detection task would decline with increasing levels of cognitive load (low, moderate, and high).

**Hypothesis two**. Higher cue utilization, as determined by participants' performance on EXPERTise 2.0, would be associated with greater accuracy in detecting phishing emails.

**Hypothesis three**. An interaction would be evident between cue utilization and cognitive load where higher cue utilization would be associated with relatively smaller reductions in performance as cognitive increased.

**Research question one**. Does a relationship exist between cue utilization groupings (higher and lower) and responses to the various cue typologies (i.e., sender's email, subject of the email, URL in the email or text in the email) when determining if an email was either trustworthy or suspicious?

**Research question two**. Does a relationship exist between decision performance groupings (high and low) on the phishing detection task and responses to the various cue typologies (i.e., sender's email, subject of the email, URL in the email or text in the email) when determining if an email was either trustworthy or suspicious?

## Methods

### Participants

Fifty adult students (35 females, 15 males) were recruited as a sample of convenience from Macquarie University's SONA research recruitment system. The participants ranged in age from 18 to 45 years (*M*_*age*_ = 20.44, *SD*_*age*_ = 4.38). The mean age for males was 21.07 (*SD* = 4.21) and the mean age for females was 20.17 (*SD* = 4.48). All participants were naïve to the context of professional cybersecurity and informed that they were participating in a study exploring how email users utilize cues to detect phishing threats under conditions of high workload. In return for their participation, students received course credit. Voluntary informed consent was obtained from all, with the research being approved by Macquarie University's Human Research Ethics Committee.

### Materials

#### Expert Intensive Skills Evaluation (EXPERTise) Program Version 2.0

EXPERTise is an online platform that consists of a battery of tests, each based on empirical investigations of cue utilization. The different tasks have been individually and collectively associated with differences in performance at an operational level (Loveday et al., 2013b). Test–retest reliability (κ = 0.59, *p* < 0.05) has been demonstrated with power control operators at 6 months intervals (Loveday et al., 2014) and with audiologists at 18 months intervals (Watkinson et al., 2018).

As cue-based associations are highly contextualized, domain-specific phishing stimuli were created for each of the EXPERTise tasks. For instance, most tasks presented users with images of emails, some of which held features that may be predictive of phishing threats (e.g., sender's address, typographical errors, prompt for action, etc.). The stimuli were reviewed by a subject-matter expert in the field of cyber-security. The EXPERTise battery comprised five separate tasks.

1. The *Feature Identification Task (FIT)* included a series of 15 phishing emails. Upon viewing each email, participants were asked to select as quickly as possible whether the email was trustworthy or untrustworthy. If untrustworthy they were to click on the part of the email that aroused their suspicion. If trustworthy, they clicked on the “Trustworthy Email” icon at the bottom right hand corner of the email. Participants' optimal use of the available cues in the email would allow for the rapid identification of its relative trustworthiness. Thus, higher levels of cue utilization are associated with a faster response latency for accurate responses (Loveday et al., 2014).
2. In the *Feature Recognition Task (FRT*) participants were presented with 10 phishing emails. In contrast to the previous task, each email would appear for 1,000 ms with the subsequent screen asking the participant to determine, on the basis of the information they observed, whether the email was “trustworthy,” “untrustworthy,” or if it was “impossible to tell.” Given the restriction placed on participants' information processing, greater response accuracy is associated with higher levels of cue utilization.
3. The *Feature Association Task (FAT*) involves simultaneously presenting pairs of words for 1,000 ms that were related to cybersecurity. Participants then indicated the extent that the two terms (e.g., Email and Malware) were related on a seven-point Likert-type scale (from 1 = “Extremely unrelated” to 7 = “Extremely related”). Higher levels of cue utilization attend to be associated with greater variance in the perceived relatedness of cybersecurity terms (Morrison et al., 2013b).
4. The *Feature Discrimination Task (FDT*) asked participants to read through two unique scenarios relating to an incoming email and then decide regarding the email's legitimacy. Following their decision, participants were presented with a list of 10 features (such as the date of email, email address and lack of detail) and using a 10-point Likert-type scale (from 1 = “Not important at all” to 10 = “Extremely important”), were asked to rate the influences of these features in reaching their conclusions. Higher cue utilization is associated with greater variance within the feature-relevance ratings (Pauley et al., 2009).
5. In the *Feature Prioritization Task (FPT*) participants determined whether an email was a phish or not. Information regarding the sender and email content is broken up into different segments that are accessible by clicking on separate tabs. Clicking on a tab would reveal the relevant information (and close any tabs previously opened). Participants had 30 s to complete their search before they would be required to decide about the email. This task assesses the capacity to acquire cues from the environment in a prioritized and non-linear pattern. Individuals with lower cue utilization are more likely to select information in the sequence in which they are presented. Higher cue utilization is associated with a relatively lower proportion of menu items accessed in the sequence they were presented (Wiggins and O'Hare, 1995). The scores are presented as a ratio ranging from 0 to 1, with lower ratios indicating greater levels of cue utilization.

#### Rail Control Task

**Figure 1:**
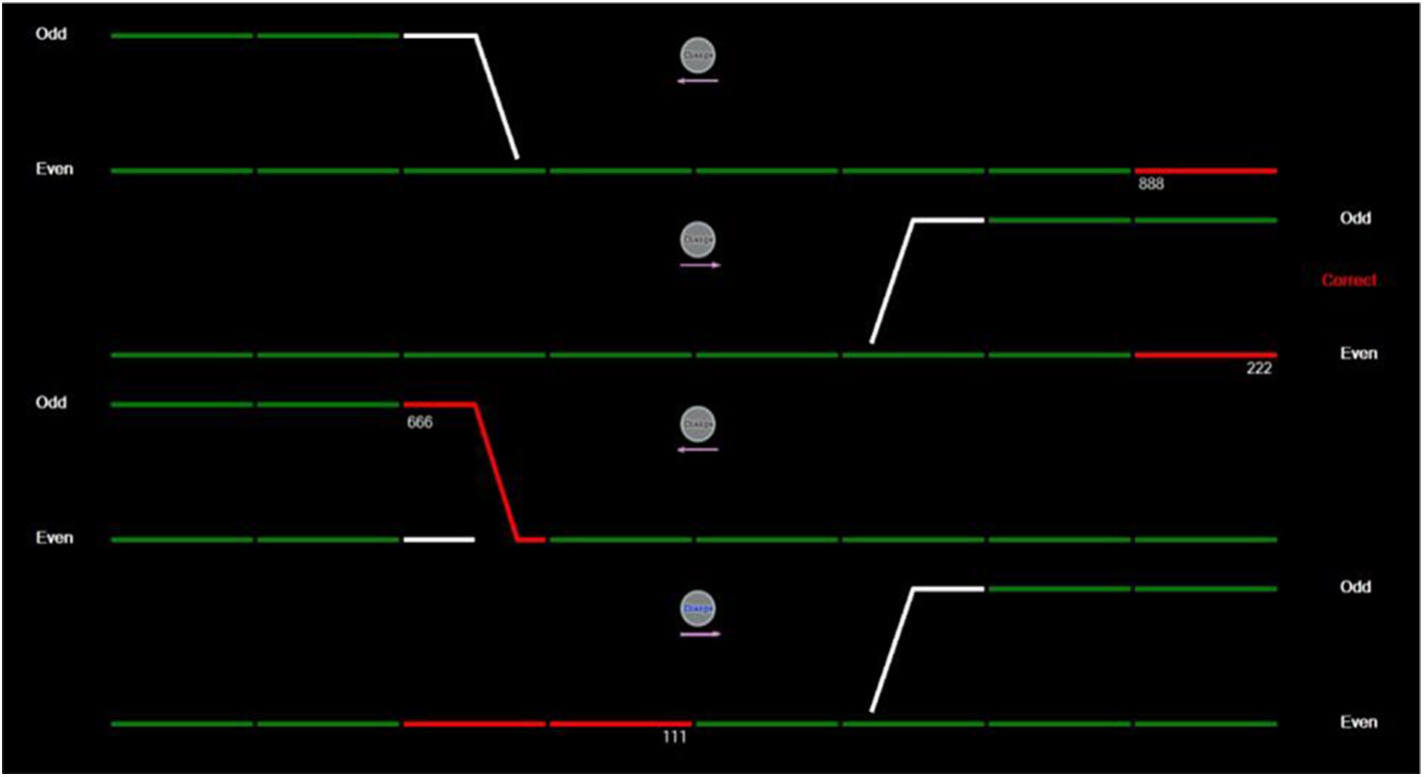
The simulated rail control task display for the high load condition.

In the rail control task, participants manage the movement of trains using a simplified simulation (example screenshot seen in Figure 1; Brouwers et al., 2017). The task consisted of four green horizontal lines that represent the railway track. Various intersections occur between these lines (depicted by white portions displayed on the tracks), with the option to change the track onto a new line. Trains are depicted as red lines and assigned either an odd or even three-digit code (e.g., 555, 888). The first and third train line run from right to left, while the second and fourth train line run from left to right. The goal is to ensure that even-numbered trains terminate on even terminals and odd-numbered trains terminate at odd terminals. To correct the programmed route of the train, participants must select the “Change” icon located above each train line. The direction of the track also appears under this icon. All trains progressed at the same speed with participants having 7 s to decide whether to re-route the train. Participants engaged three separate conditions (each comprising 21 trains), which varied in the number of train tracks being controlled at any one time. The ordering was linear, whereby cognitive load progressively increased throughout the task, which commenced with the top two train lines (low condition), then the top three train lines (moderate condition), and finally all four train lines (high condition).

#### Phishing Detection Task

**Figure 2:**
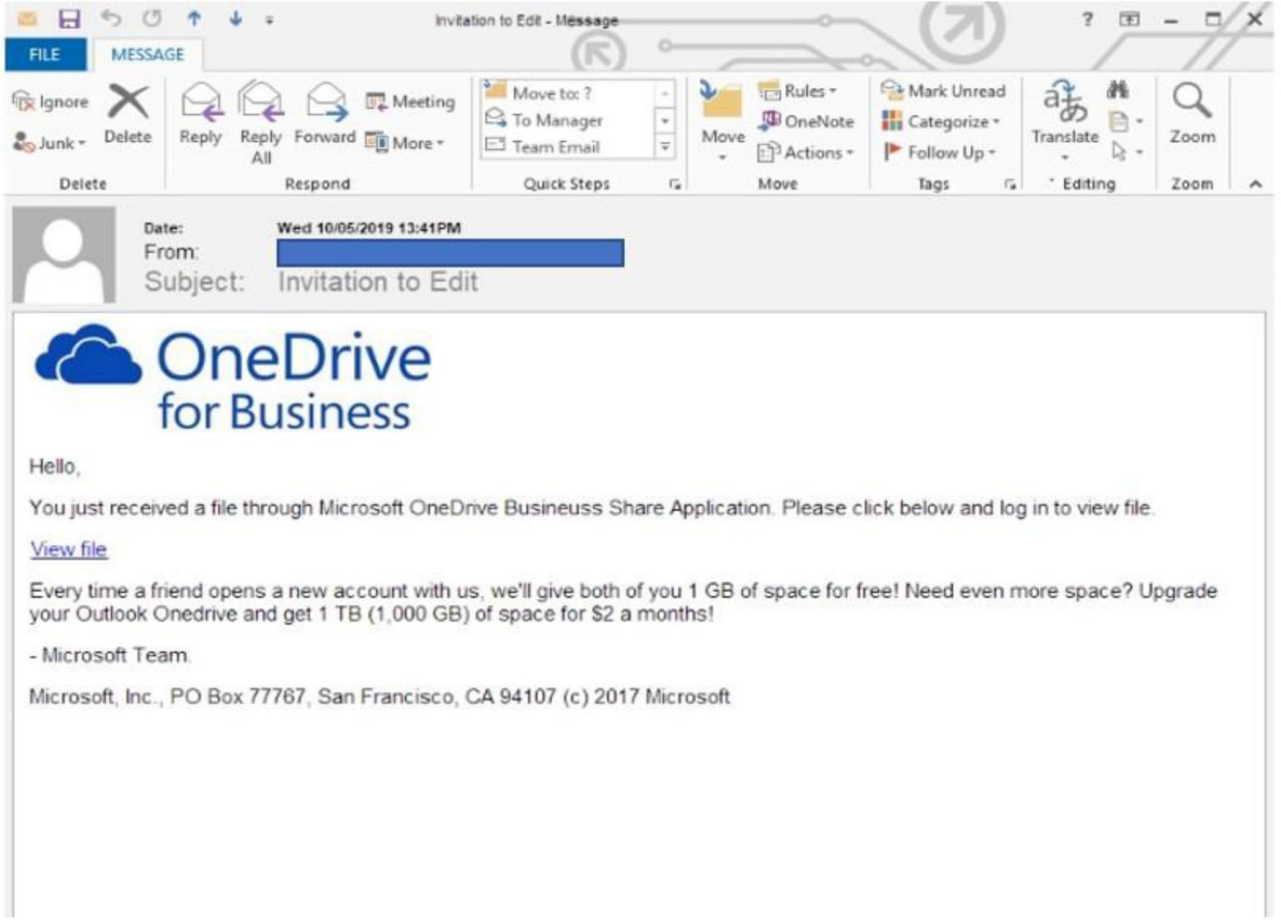
An example of a phishing email used in the phishing detection task.

Phishing emails were taken from Berkeley PhishTank and modified to an Australian context. The emails included 45 phishing emails and 45 legitimate emails (see Figure 2 for a sample phishing email). Participants responded to the emails at their own pace, and the task finished when all three conditions of the rail control task had been completed. The participants were required to respond to the emails, which varied in legitimacy as either: Trustworthy or Suspicious. After participants made a decision, they selected the Next button at the bottom of the screen, which opened a new email. This task was administered through a web-based email client simulator that was programmed to randomize the presentation of emails for each participant.

#### Task Response Feedback

This exercise consisted of two self-reported feedback items about the phishing detection task. Participants were asked to indicate from a list the cues in the email that most influenced their decisions when identifying an email as trustworthy and when identifying an email as suspicious. These responses included either the sender's email, subject of the email, URL in the email or text in the email (Williams et al., 2018).

### Apparatus

Two LG® IPSTM EA53s Desktop Monitors (24″ display size; LG Display, Yeongdeungpo District, Seoul, South Korea) were used in this experiment. The monitors connected to two Lenovo® IdeacentreTM 310S-07F (Lenovo, Quarry Bay, Hong Kong) workstations each equipped with 8GB of RAM and running a Windows 10 operating system. Each computer connected to a Microsoft® Optical wired mouse (Redmond, Washington, USA) that enabled participants to complete the tasks. The screen on the left of the participant operated the rail control task and the computer on the right of the participant operated the phishing detection task. EXPERTise operated through the same computer as the phishing detection task.

### Procedure

The participants completed the study in individual sessions of 1 h. They were seated in front of a desk with the two monitors positioned at eye level and at an approximate distance of 45 cm away from the participant. The monitor positioned on the left operated the rail control task. Prior to its commencement, participants were taken through a practice simulation of the low load condition. This task required participants to correctly re-direct an “odd” number train traveling toward an “even” numbered terminal. The second train in the practice task did not require re-directing. After completing the practice run, participants were asked if they understood the instructions. If still unsure of the task requirements, the practice task was repeated. Participants were then informed that the task would progressively increase in complexity, starting with two active train lines, then increasing to three active train lines and finishing with all four train lines active. While no specific information about the number of trains in each condition was provided, they were informed the task took 15 min to complete.

The computer screen positioned on the right of the participant was rotated into a vertical position. This position allowed participants to view and respond to the emails without having to scroll down the page. Access to the phishing detection task, required a unique URL link. Prior to completing the task, participants were instructed that they were to correctly identify the incoming emails as either “Trustworthy” or “Suspicious.” Once they had indicated a response, a “Next” button would appear at the bottom of the screen. This design allowed participants to respond to emails at their own pace. Participants were instructed not to attend to the rail control task at the expense of the phishing detection task, and that equal attention should be directed to both tasks. The task would finish at the completion of the rail control task (after 15 min), at which point they must stop responding to the email on the phishing detection task. Participants' performance on the phishing detection task contained unique timestamps information for each response. This timecode was used to match to their decision performance with each corresponding level of cognitive load.

After completing this task, participants were directed to complete a series of questionnaires on the computer screen located to their right. This process began with a task response feedback question that asked participants to indicate the cue typology they relied on when identifying emails as either trustworthy or suspicious. Finally, on the same computer, participants were instructed to complete EXPERTise, which operated through an online platform, with each of the five tasks (FIT, FAT, FDT, FPT, and FAT) accompanied by a detailed description of the task requirements on the initial screen. Participants were to independently work through the tasks and if the descriptions were unclear, to seek additional clarification from the researcher.

To avoid participants feeling that they were being scrutinized during the experiment, the researcher positioned himself in a way that prevented direct observation of their performance.

## Results

### Data Reduction

Consistent with the process outlined by Wiggins et al. (2019), EXPERTise raw scores were standardized to *z*-scores and aggregated together to create a total EXPERTise score for each participant. In preparation for a comparison of performance, a median split was employed to categorize participants as demonstrating either relatively higher or lower levels of cue utilization (Wiggins et al., 2019).

### Cue Utilization, Cognitive Load, and Phishing Detection

A 2 × 3 mixed-repeated ANOVA, incorporating two categories of cue utilization (high and low) as a between-groups variable, and three levels of cognitive load (low, moderate, and high) as a within-groups variable examined whether any significant difference existed in performance on the phishing detection task. The decision performance values on the phishing detection task were taken from the efficiency scores, which considered the number of correctly identified phishing emails as a proportion of the total number of emails to which participants responded.

**Figure 3:**
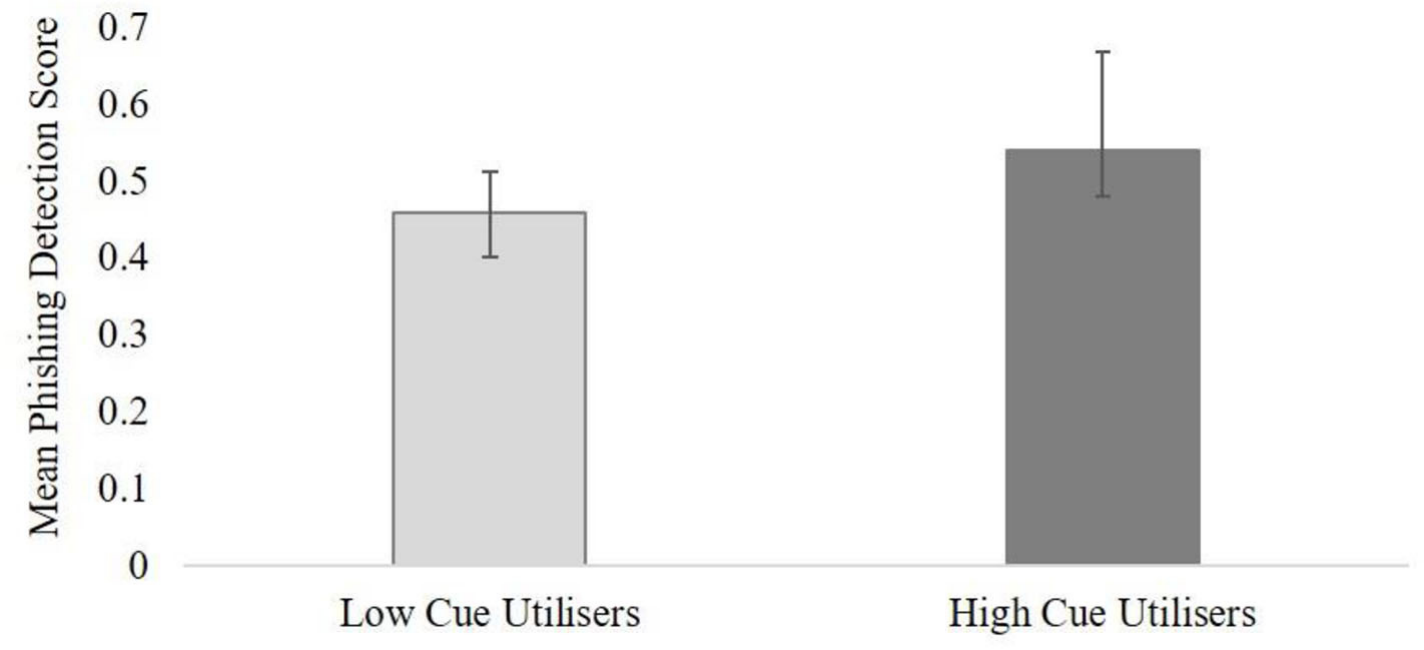
The overall mean performance for high and low cue utilization groups on the phishing detection task (mean scores are in percentages; error bars are 95% CI).

The ANOVA results revealed no main effect for cognitive load on the phishing detection task, *F*_(2, 48)_ = 2.84, *p* = 0.06 (two-tailed), η_*p*_^2^ = 0.06. As the result was in the opposite direction to our hypothesis, a decision was made not to correct the *p*-value for one-tail. This means that increases in cognitive load had no adverse impact on participants' performance during the phishing detection task and hypothesis one was not supported. The results revealed a statistically significant main effect for cue utilization, *F*_(1, 48)_ = 4.15, *p* = 0.02 (one-tailed), η_*p*_^2^ = 0.08 (medium effect), with higher cue utilization (*M* = 0.54, *SE* = 0.03) associated with greater accuracy on the phishing detection task in comparison to participants with lower cue utilization (*M* = 0.46, *SE* = 0.03) (see Figure 3). This result supported hypothesis two.

**Figure 4:**
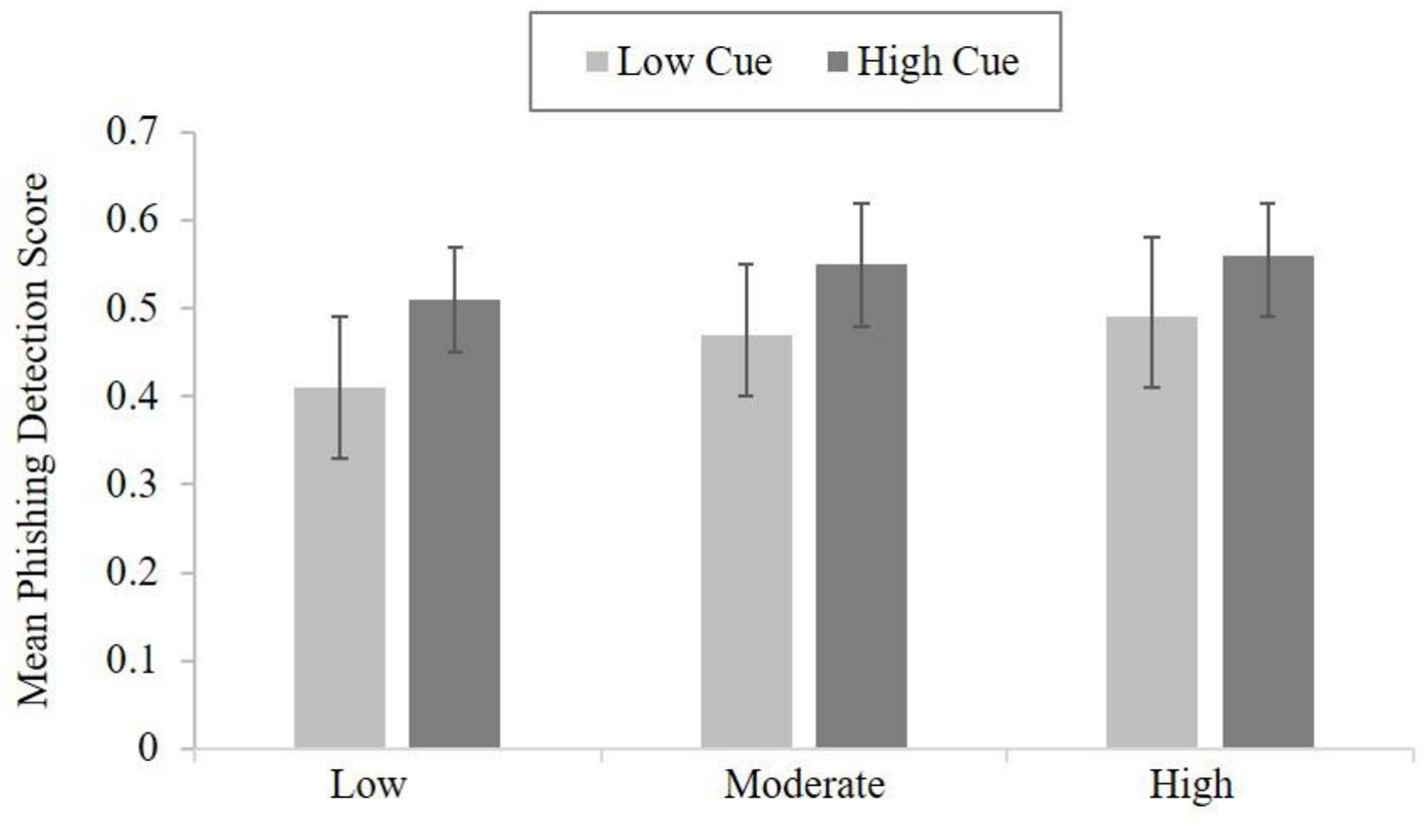
The mean performance on the phishing detection task for high and low cue utilization groups across the three levels of cognitive load (error bars are 95% CI).

As participant could respond to the emails at their own pace (and therefore, potentially manage their cognitive load via their rate of response the phishing email task), an independent *t*-test was used to test for a difference in the number of emails reviewed between the higher and lower cue utilization groups. The results did not reveal a statistically significant difference, *t*_(48)_ = −0.31, *p* = 0.761. The higher cue utilization group responded to a mean of 40.80 (*SD* = 14.60) emails and the low cue group responded to a mean of 39.50 (*SD* = 15.87) emails. Hypothesis three explored whether an interaction existed between cue utilization and cognitive load, and performance on the phishing detection task. However, the results failed to reveal any statistically significant interaction between cue utilization and cognitive load, *F*_(2, 48)_ = 0.25, *p* = 0.391. Therefore, there were no differences in accuracy based on cue utilization and accounting for differences in cognitive load (see Figure 4).

Participants were asked to indicate what features they relied on when deciding about the legitimacy of an email. They were given four cue typology options (text in the email, URL in the email, subject of the email and senders email; Williams et al., 2018) to choose from. Participants were directed to choose the cue they deemed the most trustworthy and a separate question to indicate the cue they deemed most suspicious. Two, two-way Chi-square analyses measured if a relationship existed between (1) cue utilization groups and suspicious cue typology and/or (2) between cue utilization groups and trustworthy cue typology. An examination of the assumptions for expected frequency revealed that more than 20% of the counts were <5. Therefore, any subsequent interpretation of the results must be reviewed with a degree of caution (Field, 2017).

The first two-way Chi-Square analysis revealed no significant relationship between cue utilization groups and suspicious cue typologies, $\chi_{\left(3,\;\text{N}=50\right)}^{2}$ = 1.10, *p* = 0.753. Furthermore, the second two-way Chi-Square revealed no significant relationship between cue utilization groups and trustworthy cue typology $\chi_{\left(3,\;\text{N}=50\right)}^{2}$ = 3.30, *p* = 0.349.

The following two-way Chi-square analysis focused on decision performance groups. Performance groups were derived from the mean efficiency scores on the overall task. A median split created a high (above the median) and low (below the median) decision performance groups. An examination of the assumptions for expected frequency revealed that more than 20% of the counts were <5. Therefore, any subsequent interpretation of the results must be reviewed with a degree of caution (Field, 2017). The results revealed no significant relationships between high or low performers on the phishing detection task and responses to suspicious cue typology $\chi_{\left(3,\;\text{N}\;=\;50\right)}^{2}$ = 6.13, *p* = 0.105. A final Chi-squared analysis revealed no significant relationships between high or low decision performance on the phishing detection task and trustworthy cue typology $\chi_{\left(3,\;\text{N}\;=\;50\right)}^{2}$ = 4.88, *p* = 0.299.

## Discussion

The current study tested the effects of cue utilization and cognitive load on the detection of phishing emails. The purpose was to investigate the decision-making strategies of skilled email users when formulating accurate assessments as to the legitimacy of an email.

### Cognitive Load

Contrary to the hypothesis, email users' performance on the phishing detection task was not adversely impacted by increasing levels of cognitive load (low, moderate, and high). Instead, the results indicated a trend whereby performance on the phishing task increased with each additional level of cognitive load. The observed trend may be due to a practice effect on the rail control task (Falleti et al., 2006). All participants began the task with the low load condition and progressively increased to the high condition. The initial exposure to the low load condition is likely to have familiarized participants with the task and naturally improved their performance on the subsequent conditions, despite increases in task demands. Furthermore, the improved performance suggests that the cognitive load task might not have been sufficiently challenging to disrupt participants' cognitive resources. Instead, the task may have increased participants arousal to a level that improved decision performance. Indeed, Jackson et al. (2014) explored the relationship between cognitive load, arousal and performance on a cognitive task. They found low levels of cognitive load reduced arousal and performance and that high levels of load led to an overload of cognitive resources and reduced performance (Cassady and Johnson, 2002). However, if exposed to moderate levels of cognitive load, participants arousal increased to a level that optimized decision performance on the cognitive task (Derakshan and Eysenck, 2010). Another possible explanation is that while germane load was manipulated in relation to the complexity of the task (Morrison et al., 2015), we did not assess any other measure of cognitive load. Alternative measures would give an indication of the relative load experienced by participants (e.g., pupil dilation from an eye-tracker). This data would be beneficial in establishing a more precise picture of the overall effect of cognitive load.

### Cue Utilization

Consistent with the hypothesis, higher cue utilization was associated with greater accuracy in discriminating phishing from non-phishing emails. This suggests that behavior associated with the utilization of cue-based associations in memory is associated with an increased likelihood in detecting phishing emails while undertaking a concurrent task.

These results are broadly consistent with previous research where the detection of phishing emails is presumed to be dependent upon the capacity to identify key features, such as spelling and email addresses that signify the possibility that an email is untrustworthy (Williams et al., 2018). These results are also consistent with previous editions of EXPERTise, where a greater capacity for cue utilization increased decision performance in aviation pilots (Wiggins and O'Hare, 2003), power system controllers (Loveday et al., 2013a), software engineers (Loveday et al., 2014), air traffic controllers (Wiggins and Loveday, 2015), and drivers (Brouwers et al., 2017).

### Cue Utilization, Cognitive Load, and Phishing Detection

Hypothesis three was not supported insofar as no interaction was evident between cue utilization and cognitive load. The result suggests that performance on the phishing email task was not due to differences in the capacity of participants with higher cue utilization to better manage the cognitive load associated with the rail control task, but was due possibly to an inherent capability to either recognize or maintain an awareness that enabled the discrimination of phishing from non-phishing emails (Loveday et al., 2014; Brouwers et al., 2017).

These results have implications for an explanation of phishing email detection based on an information-reduction hypothesis (Haider and Frensch, 1999). Indeed, it suggests that alternative theoretical perspectives may be involved, including the possibility that respondents are making judgements based on a template or prototype of trustworthy emails, and/or the detection of phishing emails is dependent upon a heightened level of awareness for features that characterize emails that are untrustworthy. However, an alternative explanation is that the advantage of cue utilization was not evident due to the limited number of features contained within a phishing email. Previous studies that have observed the benefits of the information-reduction hypothesis typically contain complex and dynamic environments with several task-relevant and task-irrelevant features to discriminate between (Wiggins, 2015). In Schriver et al. (2008) expert pilots were able to moderate their attention toward the most diagnostic cues when presented with a dense environment that included a range of relevant and non-relevant cues. These results were supported by Morrison et al. (2013b), where expert criminal investigators were able to decompose a complex crime scene and attend to the task-relevant cues that contained the most predictive validity to identify the unknown offender. Therefore, the narrower window of assessable features within an email means that participants might not rely on cues to minimize load since the nature of the phishing emails only requires minimal attentional resources.

### Cue Typology and Performance

The investigation into whether a relationship exists between cue utilization groups (higher and lower) or decision performance groups (high and low) and responses to the various cue typologies (i.e., sender's email, subject of the email, URL in the email or text in the email; Williams et al., 2018) resulted in no significant findings. The results indicate that when high cue utilizers were determining whether an email was trustworthy or suspicious, they were not relying on a specific phishing-related cue. Moreover, when ignoring cue utilization groups, and classifying participants by their performance on the phishing detection task, the results were the same. This seemingly indicated that all participants were considering the same cues, with the majority relying on the text within the email as a trustworthy cue. These findings support the claim that the perceptual-cognitive skill in the cybersecurity domain may be different to other areas (Brams et al., 2019). Thus, in contrast to other domains (e.g., pilots or criminal investigators; Schriver et al., 2008; Morrison et al., 2013b), skilled performance does not appear to be as reliant on the acquisition of a specific set of highly diagnostic cues. Instead, difference are seemingly due to other aspects of cue-utilization behaviors, which were effectively captured via the EXPERTise 2.0 battery. However, due to design limitations, any conclusions should be interpreted with some caution. The concept of cue utilization is associated with automatic, intuitive and unconscious processing (Klein, 1993), and asking participants to select the cues they employed from a list renders the data vulnerable to rationalization (Kelley et al., 2003).

### Limitations

In addition to the limitations discussed previously, a further notable limitation of the current work was the use of an equal number of phishing and legitimate emails in the Phishing Detection Task. Most users will receive far fewer phishing emails than legitimate ones. As such, the ratio adopted may be problematic when considering a truth-default theory in human communication (Levine, 2014). However, achieving realistic base-rates in an experimental design is challenging, as it would require participants to assess a significantly greater number of emails overall. Future studies may wish to address this limitation, as well as other experimental artifacts that may impact the generalizability of the findings to real-world environments.

Additionally, certain artifacts may have influenced the way participants engaged with the experiment (Landsberger, 1958; Finn and Jakobsson, 2007; Nichols and Maner, 2008). This included informing the participants about the research aims prior to their participation and conducting the experiment in a laboratory setting. These factors have been shown to naturally arouse suspicion levels and induce System 2 (i.e., analytical) cognitive processing (Caputo et al., 2014; Oliveira et al., 2019). Moreover, participants had no time constraints when completing the phishing detection task. The freedom provided participants ample time to assess the contents of the email, which naturally increases decision performance (Jones and Towse, 2018). Jones et al. (2019) found users are more likely to fall victim to a phishing email when under time pressure than when no time pressure was applied. The authors reasoned that time pressures forced participants to rely on their intuitive judgment, which is more prone to error. When conducting our experiment, these factors may have combined to create an artificial environment that induced more rational decision-making styles. Indeed, in naturalistic settings users typically employ System 1 (i.e., intuitive) processing when falling victim to a phishing attack (Dennis and Minas, 2018; Jones et al., 2019).

Finally, as the “low” cognitive load level was presumed to induce a negligible degree of cognitive load on participants, the study's design did not incorporate a control group (i.e., a group of participants only tasked with the phishing email task). However, it should be noted that there may still exist differences in performance based on the mere presence of secondary task, irrespective of the degree of demands placed on participants during the task. Future studies would benefit from the inclusion of such a group in their design.

### Conclusion

The current study provides an exploration of the cognitive processes associated with decision making in cybersecurity. We found an improvement in phishing email detection based on participants' degree of cue utilization. These results provide support for the proposition that the detection of phishing emails is based on the recognition of specific features that reflect untrustworthy emails. The use of cue-based training interventions has proven effective in other domains (e.g., Morrison et al., 2018), and these findings imply potential value in their adoption in the cyber-security domain.

## Data Availability Statement

The raw data supporting the conclusions of this article will be made available by the authors, without undue reservation.

## Ethics Statement

The studies involving human participants were reviewed and approved by Macquarie University Human Research Ethics Committee. The patients/participants provided their written informed consent to participate in this study.

## Author Contributions

MW, PB-S, BM, GN, and RT: conception of the work. GN, PB-S, RT, and MG: acquisition of data. GN and BM: analysis and interpretation of data. GN, BM, and MW: writing. BM: future correspondence. All authors contributed to the article and approved the submitted version.

## Conflict of Interest

The authors declare that the research was conducted in the absence of any commercial or financial relationships that could be construed as a potential conflict of interest.
